# Lipase gene fusion: a new route to chronic pancreatitis

**DOI:** 10.18632/oncotarget.5454

**Published:** 2015-09-03

**Authors:** Anders Molven, Pål R. Njølstad, Frank Ulrich Weiss

**Affiliations:** Gade Laboratory for Pathology and KG Jebsen Center for Diabetes Research, Department of Clinical Medicine, University of Bergen, Bergen, Norway

**Keywords:** Chromosome Section, chronic pancreatitis, diabetes, carboxyl-ester lipase, recombination, hybrid allele

Chronic pancreatitis is characterized by long-term progressive inflammation of the pancreatic gland, leading to permanent deterioration of its structure and to development of malabsorption, debilitating pain and diabetes mellitus. The annual incidence of the disease has been estimated to 5-10 per 100,000 persons in Western countries. The persistent pancreatic inflammation also confers a high risk for pancreas cancer, one of the major causes of cancer-related death in both men and women.

The most common explanation for chronic pancreatitis is long-term alcohol abuse, accounting for around 70% of all cases. If the disease presents as an autosomal dominant condition in a family, the term hereditary pancreatitis (HP) is often used. In 1996, a dominant loss-of-function mutation in the cationic trypsinogen gene *PRSS1* was identified as the underlying cause of HP [1]. In the two decades following this first demonstration of a genetic predisposition to chronic pancreatitis, variants in several other genes (e.g. *SPINK1*, *CTRC*, *CPA1*) [2] have been identified to increase the likelihood of disease not only in HP, but also in idiopathic (unknown cause) or even alcoholic pancreatitis. Apparently, the additive effects of environmental and inherited factors shape the ”complex disease risk” of each individual for developing chronic pancreatitis. Most pancreatitis genes discovered so far either directly encode components of the protease/antiprotease system of the exocrine pancreas or are likely to perturb this system indirectly.

**Figure 1:**
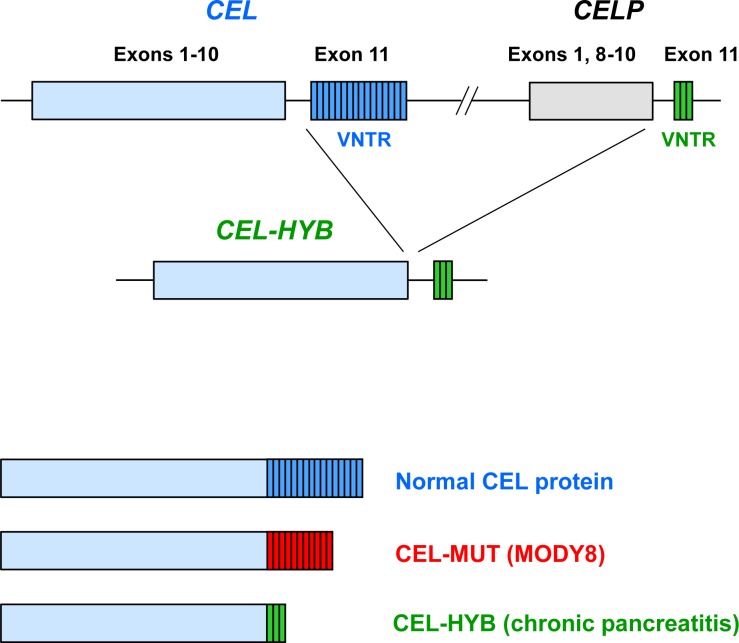
Carboxyl ester-lipase (CEL) in pancreatic disease (Top) Schematic drawing of the *CEL* gene and its adjacent pseudogene *CELP*. The latter lacks exons 2-7 but is otherwise highly similar to *CEL*. Most of exon 11 consists of a variable number of tandem repeat (VNTR) regions, each repeat being a 33-base pair segment that encodes eleven amino acids. *CEL*-*HYB* is a hybrid allele in which most of *CEL* has been fused to the VNTR of *CELP*. (Bottom) Structure of CEL protein variants. In normal CEL, the VNTR most commonly consists of 16 repeated segments. In MODY8, a single-base deletion mutation has changed the CEL C-terminal region into eleven repeats of different sequence. In the CEL-HYB variant, the C-terminal region consists of three repeats, also of altered sequence.

We have now implicated a new pathway in chronic pancreatitis by finding that a lipase-encoding gene is a significant risk factor for the disease. In a recent paper published in *Nature Genetics*, we reported that an allele of the carboxyl-ester lipase gene (*CEL*), carried by 0.5-1.0% of individuals in the general population, is more than five-fold overrepresented in subjects with idiopathic chronic pancreatitis [3]. A very intriguing twist is the way that this risk allele has arisen: it appears to stem from a non-allelic homologous recombination event between *CEL* and its neighboring pseudogene *CELP*. The result is a hybrid gene, denoted *CEL*-*HYB*, in which the proximal part stems from *CEL* and the distal part from *CELP* (Figure 1, top).

*CEL* is a remarkable gene due to its very polymorphic nature, and this is not the first time it has been linked to pancreatic disease. In 2006, we discovered that rare *CEL* mutations can cause an autosomal dominant syndrome of maturity-onset diabetes of the young (MODY) and exocrine pancreatic dysfunction, denoted MODY8 [4]. The hallmarks are exocrine insufficiency detectable in childhood, onset of diabetes in the third or fourth decade of life and development of pancreatic cysts after the age of forty [4, 5].

Although *CEL* is implicated in both MODY8 and chronic pancreatitis, the molecular mechanisms involved seem not to be identical. The last of the eleven *CEL* exons contains a variable number of tandem repeat (VNTR) region. In MODY8, a single base pair deletion has occurred in the first repeated segment, leading to a different reading frame of the C-terminus. The resulting “junk” protein (CEL-MUT, Figure 1, bottom) has altered biochemical and cellular properties, is taken up by endocytosis upon secretion, and may have a toxic effect on pancreatic cells [6]. In subjects carrying the newly discovered *CEL*-*HYB* allele, the long VNTR is exchanged with a different and shorter tail encoded by *CELP*. The resulting chimeric protein has reduced enzymatic activity compared to normal CEL, it is retained in the acinar cells and may induce an autophagic response [3]. Notably, the *CEL*-*MODY* mutations have very high penetrance and are causative for that disease, whereas *CEL*-*HYB* is a risk gene for chronic pancreatitis. In fact, most carriers of *CEL*-*HYB* in the general population stay healthy and it will be interesting to see what other factors are needed to precipitate a pancreatic inflammatory process in the susceptible individuals.

A final comment concerns the methodology. In order to map and analyze the genomic structure of *CEL*-*HYB,* we had to rely on classical molecular techniques like cloning, PCR and Sanger DNA sequencing. The *CEL*-*CELP* region is an example of a locus which is exceptionally difficult to analyze by techniques such as genome-wide association studies (GWAS) and exome sequencing. The VNTR and the presence of copy number variants including *CEL*-*HYB* make this locus extremely polymorphic and tagging SNPs that will cover the cornucopia of genetic variation in this locus are unlikely to exist [3]. In the era of high-throughput genomics and mega-consortia, it is perhaps relieving that in-depth analysis of candidate regions of our genome still can provide novel insight into human disorders.

In summary, by employing a targeted genetic approach we have revealed that a recombined hybrid allele encoding carboxyl-ester lipase is a genetic susceptibility factor for chronic pancreatitis. In the clinical routine, elevated serum lipase activity is currently used as a marker of acute pancreatitis. Interestingly, a recent study identified a genetic association of lipase activity and chronic pancreatitis with ABO blood group and fucosyltransferase-2 non-secretor status [7]. This finding and our data on *CEL* [3] indicate that lipases are not only useful diagnostic markers of pancreatic damage but that they also can play an active role in the complex risk pattern of chronic pancreatitis.
